# An Automated Text-Messaging Platform for Enhanced Retention and Data Collection in a Longitudinal Birth Cohort: Cohort Management Platform Analysis

**DOI:** 10.2196/11666

**Published:** 2019-04-02

**Authors:** Caroline M Barry, Aditi Sabhlok, Victoria C Saba, Alesha D Majors, Julia C Schechter, Erica L Levine, Martin Streicher, Gary G Bennett, Scott H Kollins, Bernard F Fuemmeler

**Affiliations:** 1 Department of Psychiatry and Behavioral Sciences Duke University Medical Center Duke University Durham, NC United States; 2 Global Digital Health Science Center Global Health Institute Duke University Durham, NC United States; 3 Cancer Prevention and Control Department of Health Behavior and Policy Virginia Commonwealth University Richmond, VA United States

**Keywords:** data collection, longitudinal studies, mobile health, text messaging

## Abstract

**Background:**

Traditional methods for recruiting and maintaining contact with participants in cohort studies include print-based correspondence, which can be unidirectional, labor intensive, and slow. Leveraging technology can substantially enhance communication, maintain engagement of study participants in cohort studies, and facilitate data collection on a range of outcomes.

**Objective:**

This paper provides an overview of the development process and design of a cohort management platform (CMP) used in the Newborn Epigenetic STudy (NEST), a large longitudinal birth cohort study.

**Methods:**

The platform uses short message service (SMS) text messaging to facilitate interactive communication with participants; it also semiautomatically performs many recruitment and retention procedures typically completed by research assistants over the course of multiple study follow-up visits.

**Results:**

Since February 2016, 302 participants have consented to enrollment in the platform and 162 have enrolled with active engagement in the system. Daily reminders are being used to help improve adherence to the study’s accelerometer wear protocol. At the time of this report, 213 participants in our follow-up study who were also registered to use the CMP were eligible for the accelerometer protocol. Preliminary data show that texters (138/213, 64.8%), when compared to nontexters (75/213, 35.2%), had significantly longer average accelerometer-wearing hours (165.6 hours, SD 56.5, vs 145.3 hours, SD 58.5, *P*=.01) when instructed to wear the devices for 1 full week.

**Conclusions:**

This platform can serve as a model for enhancing communication and engagement with longitudinal study cohorts, especially those involved in studies assessing environmental exposures.

## Introduction

### Background

Staying in contact with each other is easier today than ever before. Modern technology enables and encourages interconnectedness, overcoming the age-old obstacles of distance and time. In the past decade, cell phone use has risen globally, with the total number of mobile phone subscriptions surpassing the world population, and cloud-based apps are facilitating unprecedented, readily accessible data storage [1,2]. With these advancements saturating personal and professional networks, it follows that communication platforms would permeate health and research sectors. While studies using digital health and app-based intervention approaches to address disease self-management have boomed in recent years, there remains an apparent need to apply these approaches to clinical and population-based cohort management, particularly among large, longitudinal, community-based cohort studies [3].

Traditional methods for recruiting and maintaining contact with participants in cohort studies can be unidirectional, labor intensive, and slow. In longitudinal research studies, staff typically take various approaches to optimize study retention, including providing incentives, making repeated phone calls, sending reminder mailings, contacting family or friends, and visiting participants’ homes [4,5]. These efforts seek to maximize points of meaningful contact and keep contact information up-to-date, both important contributors to retention success [6]. Automated short message service (SMS) text messaging has been used in a number of intervention studies designed to address changes in health behaviors [2,7-14]. However, the field is lacking applied demonstrations of these methods for maintaining engagement of participants in large, longitudinal cohort studies. The following is an overview of the development process and design of a cohort management platform (CMP) used in the Newborn Epigenetic STudy (NEST). Broader applications, impact, and suggestions for future directions and use are discussed, with emphasis on the growing importance of integrating electronic cohort management strategies in health and research.

### Objectives

A CMP was initially designed and launched for use by NEST. NEST is a federally funded, longitudinal birth cohort study based at Duke University that examines how environmental exposures and nutrition, prenatally and during childhood, affect gene expression and health outcomes in children as they develop. The cohort includes 2595 mothers recruited during pregnancy between 2005 and 2011 and any children resulting from that pregnancy. The CMP launched in February 2016 and NEST participants have been recruited to enroll in the platform during study follow-up visits, pediatrician appointments, and through study mailings. The goals of the CMP with NEST are two-fold: (1) to maximize retention in this large longitudinal cohort and (2) to assist in data collection using a multimodal platform.

Maximizing retention is vital for longitudinal studies. Attrition increases with study duration and with a large cohort and accumulated years of participation, retention becomes more difficult. Participants move, contact information changes, connections fade, and longitudinal studies see increasing loss to follow-up. Loss to follow-up threatens internal validity because lost participants may be systematically different from those who stay enrolled [15,16], and high attrition can contribute to reduced statistical power [17]. The CMP aims to address this issue by enhancing outreach and facilitating a quick, convenient means of interactive communication. Participants are equipped with a service for immediate connection, which aims to boost not only the number of contact points between participants and staff, but also the likelihood of maintaining up-to-date contact information and overall rapport with the study.

The second objective of the CMP is to assist with data collection. Survey questions are delivered directly to participants, who can key in responses on their mobile devices in real time. The advantages to this method include convenience for both participants and staff, cost efficiency, and real-time responses. Real-time responding increases data quality by reducing recall burden [18,19]. Immediacy of data collection can also provide a more complete picture of exposures, thoughts, emotions, and other variables of interest as they are experienced throughout the day. The following provides a brief overview of the CMP’s development and operation, utility for NEST, strengths and limitations of the method, and broader applications for future use.

## Methods

### Development

The CMP was adapted and iteratively enhanced from a platform already in use by the Duke Global Digital Health Science Center. The platform is a flexible, cloud-based communication system designed to aid in behavioral interventions. Leveraging prior experience and existing code allowed for rapid development of the CMP at a lower cost than developing a system de novo. In earlier iterations of the platform, both the interactive SMS and interactive voice response (IVR) components contributed to high rates of engagement. Thus, those interactive features became central to the CMP.

The CMP runs on a custom-built, integrated, automated, interactive digital health system. Third-party vendors, such as Twilio, Heroku, Mandrill, and Amazon S3, provide relatively inexpensive infrastructure to send and receive messages from participants. Messaging within the CMP reads specific participant-tracking data fields, such as dates (eg, date of birth and date of last study visit), and delivers messages on a predetermined schedule using a customized library developed by the study team. For example, one message is sent to wish participants a happy birthday. Another message reminds participants to perform a task and yet another reminds participants about upcoming appointments. When appropriate, messages are further personalized to each participant to include the mother's and/or children’s names. Daily messages sent over a 1-week period remind mothers to have their child, who is referred to by name, wear an accelerometer to record physical activity.

**Figure 1:**
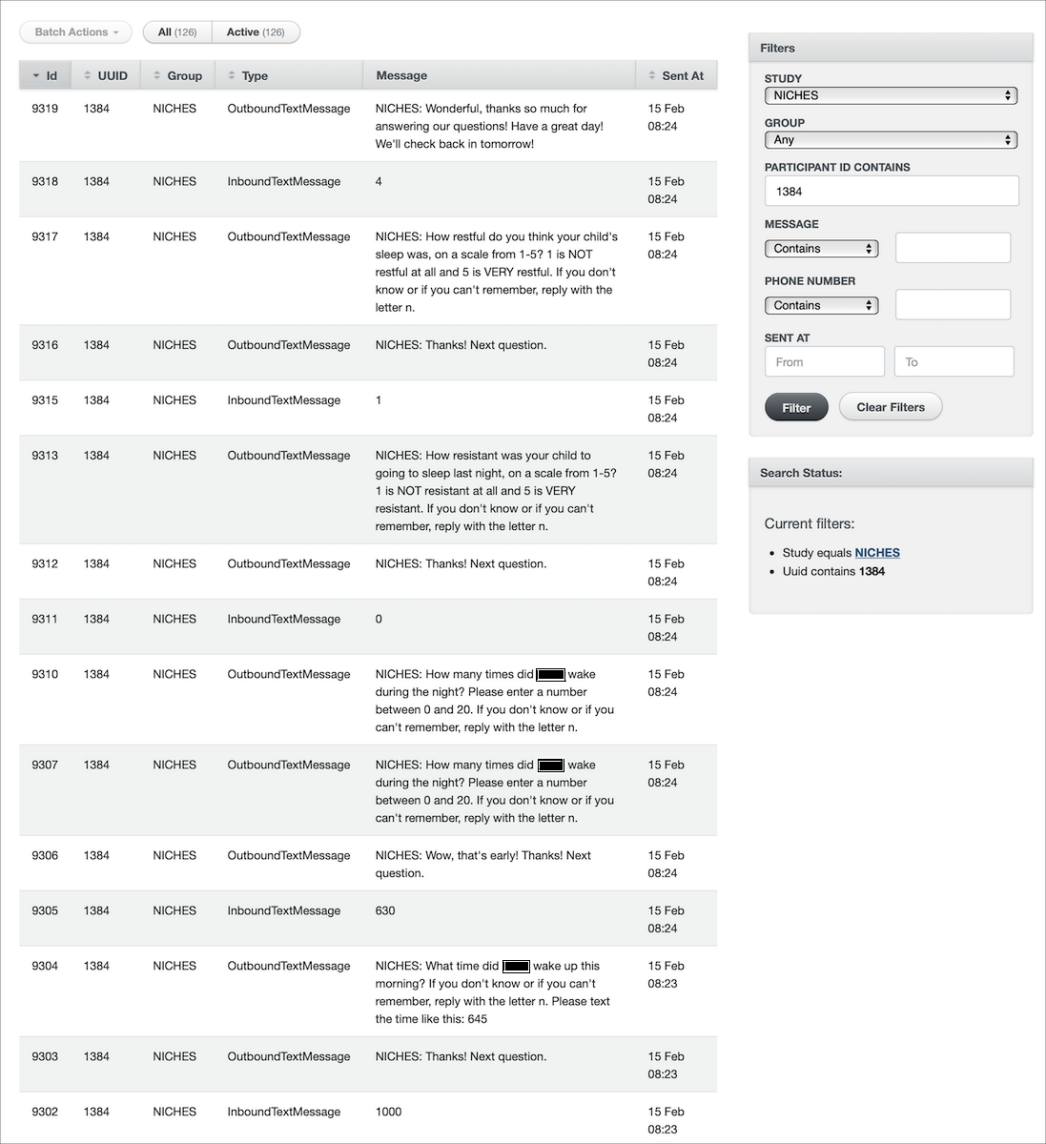
Researcher dashboard of the cohort management platform (CMP).

The platform also provides simple bidirectional messaging, where participants may respond to platform-generated text message queries. For instance, a message delivered in the morning asks participants to reply with qualitative measures of their child’s sleep quality and duration the night before.

A back-end dashboard (see Figure 1) was also built to allow the research team to review and monitor, in real time, when and what types of messages are sent and received from the participants. The study team is able to export data from the platform and merge it with central study databases for analysis. The study team also uses this back-end dashboard to register participants in the CMP once consent is given.

### Consent

The CMP has been thoroughly reviewed by the Duke University’s Office of Clinical Research and Institutional Review Board for data security and integrity and to ensure ethical use. The CMP uses third-party vendors, such as Twilio, Heroku, Mandrill, and Amazon S3, to send messages, which encrypt participant information on their servers. However, if these companies or auditors opt to share these data, federal privacy protections may no longer apply. Further, it is recognized that no electronic communication channel is completely secure and confidential. The consent process involves informing participants who enroll to receive messages through the CMP of these potential risks; participants are given the option to decline enrollment in the CMP in favor of traditional communication methods while still remaining in the study.

## Results

### Initial Participant Engagement

There were 302 NEST participants who were approached for CMP consent: 56.6% (171/302) were non-Hispanic black, 35.8% (108/302) were non-Hispanic white, 3.0% (9/302) were Hispanic, and 4.6% (14/302) were categorized as *other*. The mean age of 283 respondents was 33.9 years (SD 5.7). The mean annual household income of 278 respondents was US $56,697 (SD US $63,452). Since February 2016, of the 302 NEST participants who were approached for CMP consent, 98.7% (298/302) agreed to use the system and provided consent while 1.3% (4/302) opted out. A total of 162 participants are actively registered and using the system (ie, responded to a CMP message). Prior to registration of participants to the CMP, participants were informed of the risk and consent was obtained by study staff during an in-person study visit. Study staff then registered participants in the system using the back-end platform and shortly thereafter they received a *welcome* message. Participants then received the series of scheduled messages and were asked to interact with the system’s short queries when prompted. Participants were given the option of withdrawing at any time, which they could do through the system by sending a message saying “stop.” None have withdrawn thus far.

Although the CMP currently employs SMS text messaging as the primary method of communication, the study team has been asking participants, for quality improvement, to indicate how they would like to receive messages (ie, SMS, email, and/or IVR). Multiple responses are possible. Of those who have consented thus far, the majority agreed to receive text messages (266/302, 88.1%) and/or emails (278/302, 92.1%); fewer chose IVR (68/302, 22.5%). Given this initial success and that many participants chose more than one communication channel, our next step will be to roll out email channels followed by IVR to actively engage with the remaining participants for longitudinal follow-up.

### Benefits to Study Management

Overall, study management and data collection for specific measures has been enhanced by using the CMP. For instance, some of the communication from study staff to participants regarding appointments can be managed through the CMP. Also, during the period when participants are asked to have their child wear their accelerometers, automated reminders are being sent and, at the same time, participants are being queried about their child’s sleep duration and quality via automated text. Any unanswered text messages can be resent repeatedly on a designated schedule to improve data capture. Performing these same procedures with live study staff would result in a greater burden on the study team. For example, reminding participants to have their child wear the accelerometer and to inquire about their child’s sleep would require a staff member to make a phone contact with participants every day. For the 302 participants, one 3-minute call every day for 7 days adds up to 105.7 hours per week. Off-loading this to the CMP frees up time from staff to perform other vital tasks.

### Enhancement of Data Collection and Cohort Retention

**Figure 2:**
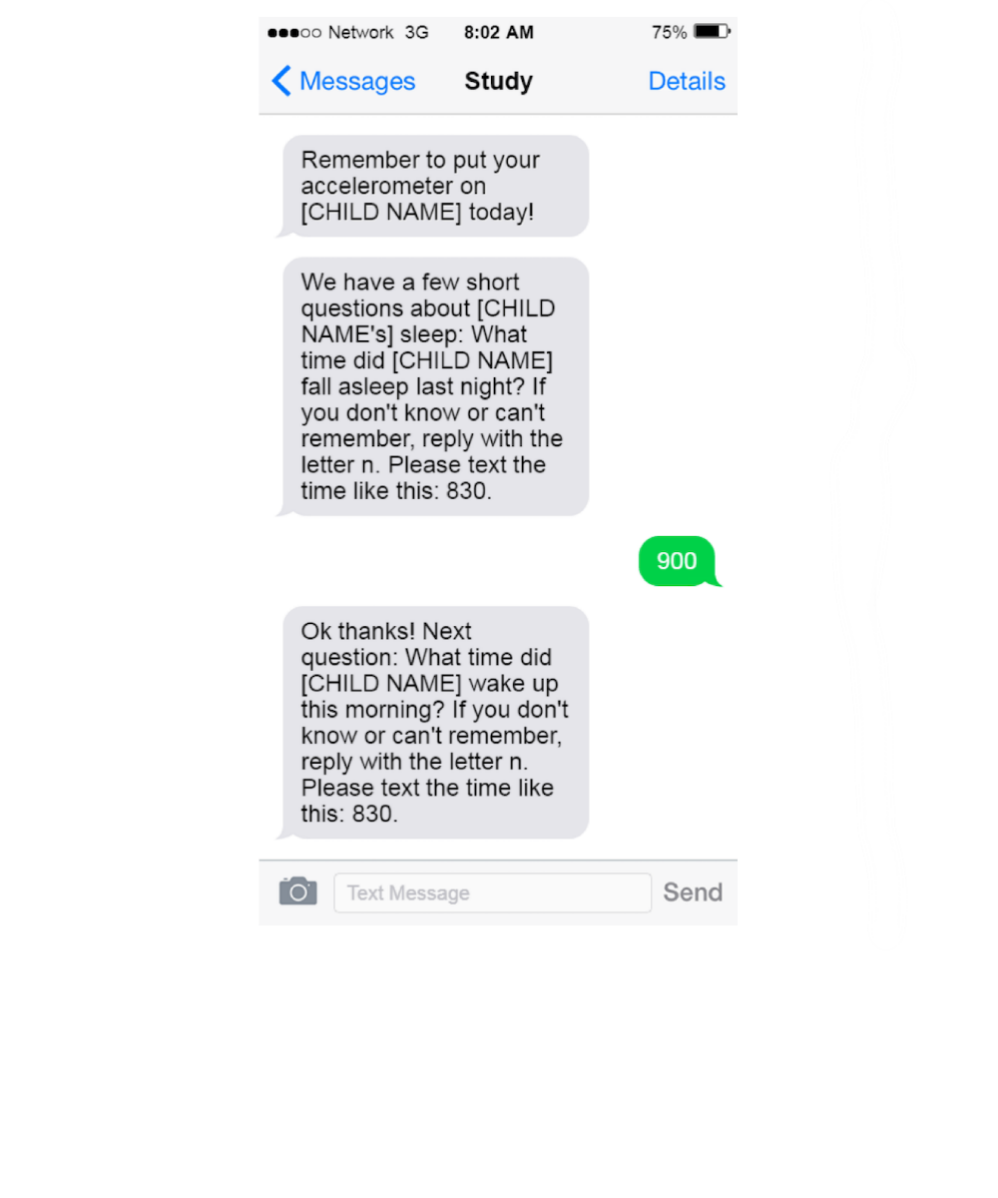
Successful response to the cohort management platform (CMP).

**Figure 3:**
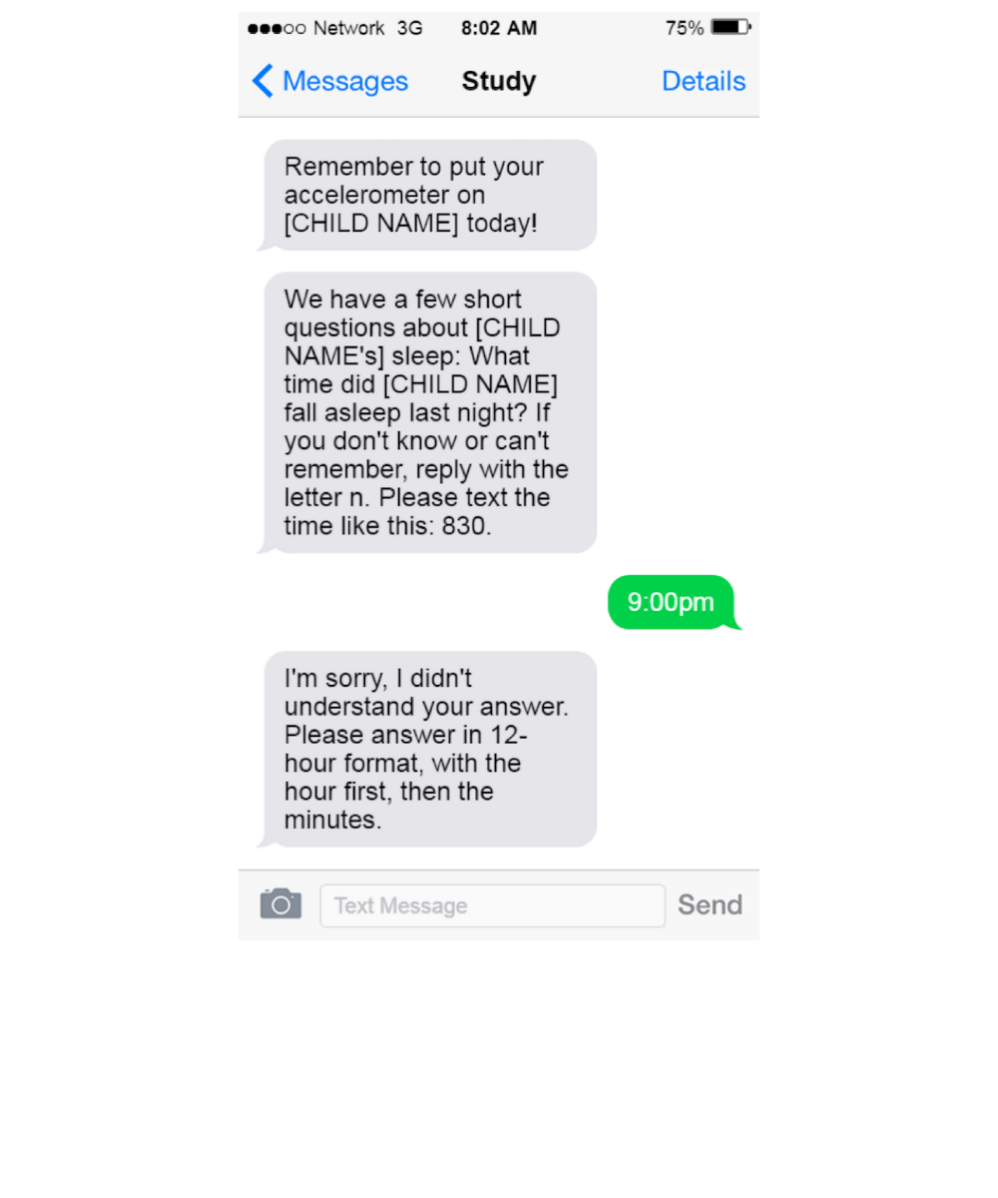
Unsuccessful response to the cohort management platform (CMP).

Beyond these methods of communication, the platform is used for data collection. Currently, the CMP is being used to collect data in two NEST follow-up studies. One study is designed to have two in-office appointments that are separated by no more than two weeks; during the interim, self-reported sleep data are gathered from participants using the CMP. The platform sends short text message queries asking participants to note their child’s sleep duration and quality. As mentioned above, these questions are sent each morning, which gives mothers the ability to immediately reply, decreasing their burden to record or remember data from each night of sleep. In addition, the platform also sends participants daily reminders to have their child wear his or her accelerometer between their two visits. Initial data suggests that this is helping to increase adherence and maximize available data. For example, each morning at a preset time, typically 8 AM unless otherwise indicated, a reminder is sent to put the accelerometer on themselves and their child. The participant then receives questions about their child’s sleep quality. The CMP waits for a valid response (ie, any 3 or 4 digits following a time pattern) and, if valid, the response is saved and the CMP sends the next question (see Figure 2). If the response is not valid, the CMP sends a prompt to try again (see Figure 3).

Preliminary data show that the CMP messaging may be helping to improve adherence to the accelerometer protocol. At the time of this report, 213 participants in our follow-up study who were also registered to use the CMP were eligible for the accelerometer protocol. This involves asking mothers to have their child wear an accelerometer on a daily basis for at least 7 days. Participants were told that they would be receiving text messages via the CMP to remind them to have their child wear his or her accelerometer. Of these 213 participants, we categorized them into *texters* and *nontexters,* based on whether they responded to a text at any point during the use of the CMP. Texters (138/213, 64.8%), when compared to nontexters (75/213, 35.2%), had significantly longer average accelerometer-wearing hours (165.6 hours, SD 56.5, vs 145.3 hours, SD 58.5, *P*=.01).

**Figure 4:**
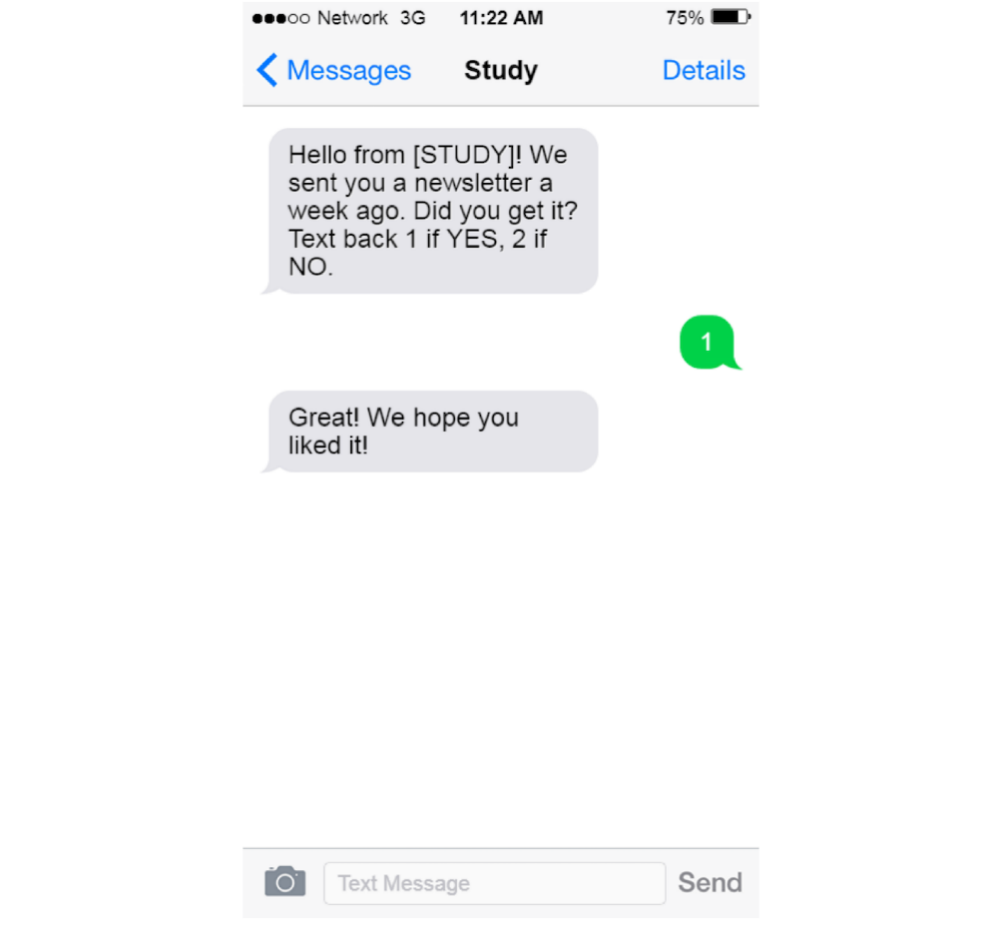
Response to the cohort management platform (CMP) indicating that the newsletter was received.

To enhance cohort retention, the CMP is being used to verify participant mailing addresses. For instance, messages are being sent after batch newsletters, and recruitment letters are mailed to confirm that they received these mailings (see Figure 4). If receipt is confirmed, the CMP thanks the participant; if not, the CMP requests updated contact information. New addresses can then be automatically fed into the study database to update records.

## Discussion

### Impact

To date, the CMP is showing great potential for maximizing retention and improving data collection among a large, longitudinal, community-based cohort. It is being used to address the inefficiency of traditional unidirectional participant contact protocols in numerous ways, such as the following: (1) by cutting down on staff time and effort dedicated to phone calls, mailings, and appointments; (2) by reducing resources for paper mailings and the waste of returned mail; and (3) by diminishing the burden placed on participants for measures requiring daily diaries.

Staff time and effort typically devoted to individual phone calls and single mailings can be curtailed with the aid of systems like the CMP because it capitalizes on automation while retaining personalization capacity for tailored messaging. Paper saved using the CMP is itself a compelling advantage, with immediate resource-saving and eventual positive environmental impact. Further, the CMP allows for data collection in near real time. This can be particularly useful since measurement can be timed to be contemporaneous with key behaviors as they occur during the participant’s daily life (eg, asking participants in the morning when they went to sleep the night before and when they woke up). In general, shortening the recall period for certain behaviors, such as sleep, improves accuracy [18,19]. In addition, it can be a lot easier for participants to respond quickly to a text message than to fill out a paper-based log and the data is captured automatically, thereby avoiding the need for staff to manually enter such data.

### Strengths and Limitations

Strengths of the CMP include its potential cost-effectiveness, time-effectiveness, timed delivery of survey questions, real-time receipt of responses, and its accessibility through commonly used communication channels (ie, SMS text messaging). Unlike alternative data collection tools, such as REDCap or Qualtrics, the CMP is not a database platform, although it can be used in conjunction with them. Database platforms rely primarily on manual data entry, whereas the CMP can run independently of staff to automate data collection and make real-time decisions and actions based on its collected data. Further, unlike REDCap or Qualtrics, the CMP is unique in its capacity to trigger the delivery of highly tailored messaging to perform a variety of tasks (eg, greetings, reminders, contact information changes, and data collection) over predetermined time periods. Importantly, the CMP can be used together with database platforms to achieve common goals of longitudinal cohort management. In general, the CMP can serve as a model for implementation of analogous designs.

Of note, there are some limitations of the data presented here as well as the CMP system. As it pertains to this report, we did not collect any data on why participants opted out of the CMP, which could be useful to better understand how to increase cohort management methods that are inclusive to all participants. Fortunately, for this particular sample, only around 1% of participants opted out of using the CMP, thus demonstrating that this type of system may be fairly acceptable to participants. Another limitation pertaining to the data presented here is that although we showed that compared to nontexters, texters had greater adherence to the accelerometer wear protocol, this does not confirm improved adherence in favor of use of the platform. Future studies using a rigorous randomized design are needed to fully evaluate the benefits of this type of reminder system on participant protocol adherence. A current limitation of the CMP system is that the short message queries are time sensitive. If a participant does not respond to a query within a predetermined time period, they will have missed the opportunity to provide their response and cannot respond to the query at a later time. This can be overcome by increasing the overall period of monitoring (eg, from 7 days to 14 days) to ensure sufficient data across an acceptable number of days. Finally, a limitation of using automated messaging systems in general may be the impersonal nature of the communication, which may have the unintended consequence of decreasing positive affiliation among participants to the cohort study. To guard against this, our approach has been to view the CMP as an ancillary tool to other methods we are using to make participation in the study more personal. We continue to reach out to participants via newsletters and one-on-one communication when necessary.

### Future Directions

Future uses of the CMP with our particular study include increasing the number of registered participants in the system, continued recurring automated exports from the study database with participant information to the platform, regular birthday and holiday greetings, appointment reminders, integration of other data collection instruments, and promotion of study newsletters and publications. The development team is also working on integrating other modes of communication into the platform, such as building in methods that connect with Facebook and Twitter. Additionally, the team has begun to develop methods to capture data from commercial activity trackers, such as Fitbit, and linking the system with Apple’s ResearchKit and Google Fit, which would allow for capturing additional health tracking data.

From an epidemiology study perspective, the CMP has the potential to cast a wider net in the collection of exposure data. Participants, equipped and comfortable with tools for immediate reporting, may selectively attend to previously overlooked or misestimated exposures. Further, the CMP could aid in research assessing physical health factors (eg, weight, physical activity, sleep, and smoking) and mental or emotional variables (eg, depressive symptoms and stress). Participants could self-report on these health outcomes to provide real-time updates and avoid the cost and time of in-person appointments, thereby boosting data quantity and quality and limiting participant burden. Although the CMP is also used for tracking diet and physical activity in obesity interventions [7,9], it could be further utilized to monitor diet and activity in observational longitudinal cohort or clinical studies. In addition, as mentioned above, future iterations will allow for capturing commercial sensor and health tracking data that could be used to enrich understanding of the myriad of factors that play a role in health and wellness.

### Conclusions

The CMP can serve as a model for enhancing interactive communication and data collection with longitudinal study cohorts, as it takes advantage of modern technology and keeps pace with participants’ changing communication preferences. Ease, accessibility, and points of contact increase with the help of the CMP and it facilitates more opportunities for exposure assessment. Continued implementation research is needed to advance technologies like the CMP, though its potential as a longitudinal research tool is highly promising.

## Abbreviations

CMP: cohort management platform
IVR: interactive voice response
NEST: Newborn Epigenetic STudy
NIH: National Institutes of Health
SMS: short message service
USEPA: United States Environmental Protection Agency
